# Oxytocin modulates social preference and anxiety-like locomotor behavior in albino and non-albino zebrafish

**DOI:** 10.17305/bb.2026.13819

**Published:** 2026-04-09

**Authors:** Ana-Maria Danila, Alexandra Savuca, Mircea-Nicusor Nicoara, Alin-Stelian Ciobica, Bogdan Novac, Stefan Chiriac, Daniela Tomita, Ovidiu-Dumitru Ilie

**Affiliations:** 1 Department of Biology, Faculty of Biology, Alexandru Ioan Cuza University of Iasi, Iasi, Romania; 2 Ioan Haulica Institute, Apollonia University, Iasi, Romania; 3 Olga Necrasov Center, Biomedical Research Group, Romanian Academy, Iasi Branch, Iasi, Romania; 4 Academy of Romanian Scientists, Bucharest, Romania; 5 CENEMED Platform for Interdisciplinary Research, University of Medicine and Pharmacy, “Grigore T. Popa”, Iasi, Romania; 6 Faculty of Medicine, Grigore T. Popa University of Medicine and Pharmacy, Iasi, Romania; 7 Clinical Department, Apollonia University, Iasi, Romania; 8 Regional Center of Advanced Research for Emerging Diseases, Zoonoses and Food Safety, Department of Public Health, Faculty of Veterinary Medicine, Ion Ionescu de la Brad Iasi University of Life Sciences, Iasi, Romania

**Keywords:** Oxytocin, albino, zebrafish, social, behavior, anxiety.

## Abstract

Socio-emotional and exploratory behaviors in zebrafish (*Danio rerio*) are shaped by conserved neuromodulators such as oxytocin (OXT), yet phenotypic variation (e.g., albinism) may modify sensitivity to OXT-dependent regulation. Here, we tested whether pigmentation phenotype alters behavioral responses to acute OXT across structured social and anxiogenic contexts. Adult albino (A; *n* ═ 30) and non-albino (N; *n* ═ 30) zebrafish (total *n* ═ 60; equal allocation across 6 groups, *n* ═ 10/group) were exposed to OXT by immersion (33.2 ng/mL, 15 min) under a 2 × 3 factorial design (Phenotype × Dosing regimen: Vehicle; OXT single exposure assessed at 24 h; OXT repeated exposure assessed at 48 h) and evaluated using the social preference test (SPT), social interaction (SI) test, and novel tank test (NTT). In the SPT, decision-zone occupancy and mean distance to the non-social arm were robustly modulated by dosing (*P <* 0.0001; *P ═* 0.007) without phenotype × dosing interactions; locomotor outputs (swimming distance, *P ═* 0.040; velocity, *P ═* 0.041; counterclockwise rotations, *P ═* 0.006) also showed dosing effects only, supporting a dissociation between social allocation and global motor output. In the SI test, stimulus-zone occupancy showed a significant main effect of phenotype (*P ═* 0.009), independent of dosing, and locomotor indices similarly exhibited phenotype effects without interaction (swimming distance and velocity, *P ═* 0.017; mobile-state duration, *P ═* 0.002). In contrast, the NTT revealed phenotype-dependent locomotor regulation: highly mobile duration showed a significant interaction (*P ═* 0.002) with strong main effects of phenotype and dosing (both *P <* 0.001), while immobile duration displayed an interaction (*P ═* 0.015) and dosing effect (*P ═* 0.005), driven primarily by the non-albino phenotype; clockwise rotation (*P ═* 0.018) and active-state duration (*P ═* 0.030) further indicated phenotype-related differences. Collectively, OXT exerts context- and dosing-regimen-dependent modulation in zebrafish, with social allocation primarily dosing-driven and largely phenotype-independent, whereas anxiety-related locomotor dynamics show marked phenotype specificity.

## Introduction

Albinism is a rare genetic condition characterized by impaired melanin production, with effects that extend beyond visible hypopigmentation. Traditionally considered a pigmentary disorder, emerging evidence indicates that albinism has broader implications, particularly regarding physical manifestations that influence social perception, behavioral regulation, and neurochemical processes. Consequently, studying albinism offers a valuable framework for understanding how genetic and phenotypic variations can shape socio-emotional responses across species [1].

Albinism encompasses a heterogeneous group of inherited disorders caused by mutations in genes involved in melanin biosynthesis within melanocytes, the cells responsible for pigmentation [2, 3]. Clinical forms are broadly classified into oculocutaneous albinism (OCA), affecting the skin, hair, and eyes, and ocular albinism (OA), which primarily targets the visual system, depending on the affected gene and the degree of melanin reduction [2–5].

From a genetic perspective, albinism represents a significant public health concern due to its medical and psychosocial effects. Its prevalence varies geographically, affecting approximately 1 in 17,000 to 20,000 individuals in Europe and the United States (US), while reaching up to 1 in 1,000 in certain African populations, influenced in part by consanguinity and limited geographic mobility [6–8]. Notably, individuals with albinism are affected regardless of their social class. Psychosocially, they often face stigmatization, social isolation, and anxiety, factors that can impede interpersonal relationships and social integration [9–11].

Phenotypic variation may interact with neurobiological systems involved in emotional and social regulation. One notable network is the oxytocinergic system, a highly conserved neuropeptide in vertebrates that plays a central role in regulating social behavior, stress reactivity, and exploratory activity [12, 13]. In mammals, OXT is implicated in mother-infant bonding, pair bonding, social recognition, trust, and empathy [14–16].

Although OXT is frequently associated with prosocial and anxiolytic effects, evidence from zebrafish and mammalian studies suggests that its behavioral impact is context-dependent. The influence of OXT on social engagement and anxiety-like behavior may vary according to environmental conditions, baseline phenotypes, and treatment parameters, with some studies reporting contrasting or phenotype-specific effects [17]. Recent findings further indicate that OXT can selectively modulate behavioral responses based on phenotype, influencing social motivation and exploratory activity in both social and anxiogenic contexts [18].

In this context, zebrafish (*Danio rerio*) serve as an ideal experimental model for investigating how genetic and phenotypic variation influences OXT-mediated behavioral regulation [19, 20]. Animal models are essential for understanding the genetic, molecular, and behavioral mechanisms underlying albinism, and zebrafish have emerged as one of the most valuable vertebrate models for studying pigment-related mutations and their behavioral consequences. Albino zebrafish exhibit mutations in genes critical for melanophore development and melanin synthesis, including melanocyte inducing transcription factor (MITF), tyrosinase (TYR), dopachrome tautomerase (DCT), and OCA2 melanosomal transmembrane protein (OCA2), which correspond to human homologs [21–23].

In addition to their genetic tractability, zebrafish offer a robust and quantifiable behavioral platform [24, 25]. Their social and exploratory behaviors can be reliably assessed using standardized paradigms, such as the social interaction (SI) test, social preference test (SPT), and novel tank diving test (NTT) [26, 27]. These paradigms provide validated measures of social affiliation, exploratory drive, and anxiety-like behavior. The integration of genetic conservation, behavioral sensitivity, and neurochemical accessibility establishes zebrafish as an exceptionally suitable model for exploring the interaction between pigment-related mutations, neuromodulatory plasticity, and behavioral outcomes [28, 29].

Overall, the present study aims to investigate phenotype-dependent behavioral differences in zebrafish under OXT treatment. Albino and non-albino fish were evaluated using standardized paradigms assessing social, exploratory, and anxiety-like responses. By integrating genetic phenotype with neurochemical modulation, this study seeks to clarify whether OXT differentially regulates behavior as a function of pigment-related phenotype, thereby highlighting the potential influence of albinism on socio-emotional reactivity and neuromodulatory mechanisms.

## Materials and methods

### Ethical statement

All experimental procedures were conducted in accordance with the European Commission Recommendation (2007) and Directive 2010/63/EU of the European Parliament and of the Council (22 September 2010) on the protection of animals used for scientific purposes [30, 31]. The study protocol was approved by the Ethics Committee of the Faculty of Biology, “Alexandru Ioan Cuza” University of Iasi, under registration number 1349/20 March 2024.

### Animal maintenance

A total of 60 adult zebrafish (*Danio rerio*) were used in the study, comprising 30 non-albino (N) and 30 albino (A) individuals. The fish were obtained from an authorized breeder (Aqua Planet, Bucharest, Romania) and were approximately 6 months old at the start of the experiment. The animals underwent a three-week acclimation period in 10 L aquaria equipped with oxygen pumps and daily water changes. During acclimation and throughout the experiment, water temperature was maintained at 26 ± 1 ^∘^C, pH at 7.0–7.5, and a 14 h light/10 h dark photoperiod was applied. Salinity was maintained at 0.5 ppt, conductivity at 500–600 µS/cm, and ammonia levels remained below 0.01 mg/L. After acclimation, zebrafish were randomly assigned to experimental groups (*n* ═ 10 per group). Although sex distribution was not matched, both males and females were represented in all experimental groups. All procedures were performed in accordance with the updated Animal Research: Reporting of *In Vivo* Experiments guideline (ARRIVE 2.0) [32] and the principles of the 3Rs [33] to ensure animal welfare.

### Experimental design

Zebrafish were randomly assigned to experimental groups following acclimation under the conditions previously described. Six experimental groups were established (*n* ═ 10 per group): Albino vehicle control (CTR A), Albino OXT 24 h (A 24h), Albino OXT 48 h (A 48h), Normal vehicle control (CTR N), Normal OXT 24 h (N 24h), and Normal OXT 48 h (N 48h). Pharmaceutical-grade oxytocin (OXT) sourced from Pasteur Company, Bucharest, Romania, at a concentration of 10 I.U./mL, was diluted in system water to achieve a final concentration of 33.2 ng/mL. This concentration was selected based on previous studies [34, 35] demonstrating effective modulation of social, exploratory, and oxidative stress-related behaviors in adult zebrafish. Fish were exposed collectively within their respective tanks to the OXT solution for 15 min.

**Figure 1.**
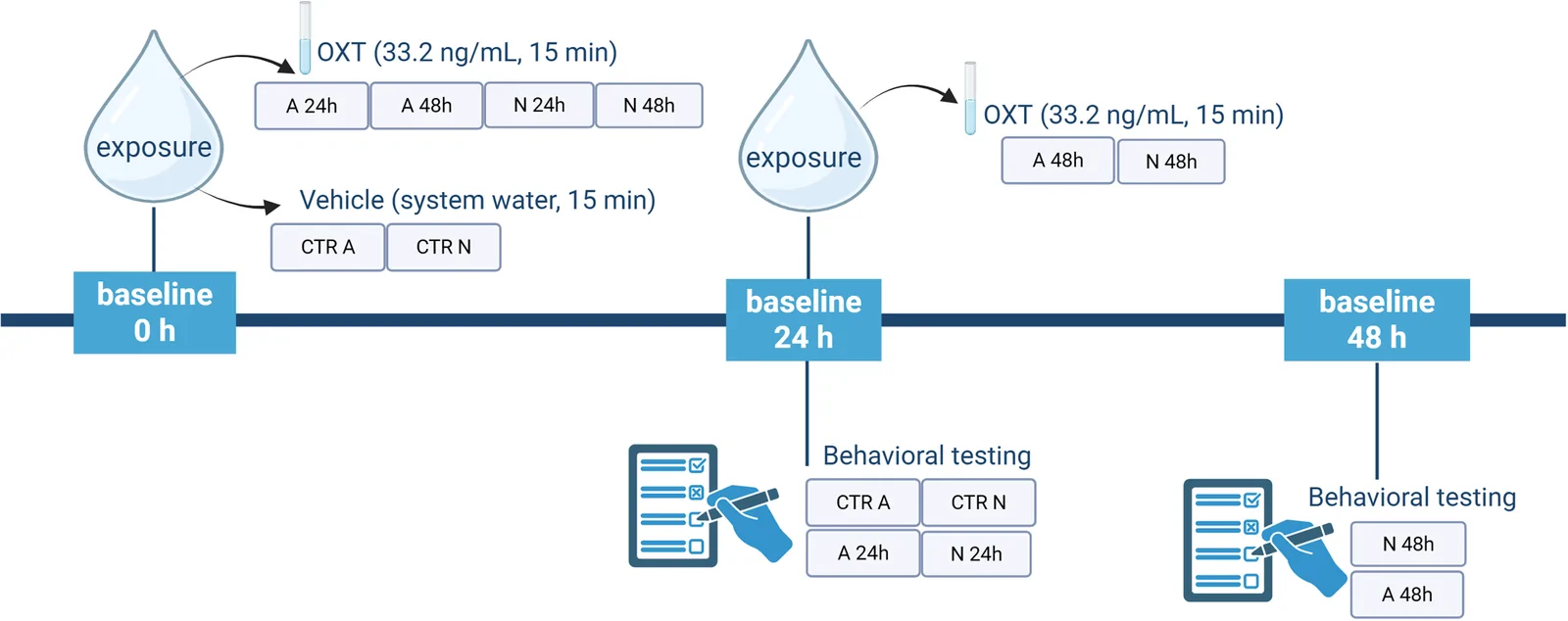
**Timeline of zebrafish exposure and behavioral testing.** Six experimental groups were analyzed: Albino vehicle (CTR A), albino OXT 24 h (A 24h), albino OXT 48 h (A 48h), normal vehicle (CTR N), normal OXT 24 h (N 24h), and normal OXT 48 h (N 48h). Behavioral testing for the vehicle and OXT 24-hour groups was conducted at 24 h, while the OXT 48-hour groups underwent a second OXT exposure. Behavioral assessments for the OXT 48-hour groups were performed at 48 h. **Abbreviations:** CTR, control; OXT, oxytocin.

Control (CTR) groups were handled identically to treated groups and were exposed for 15 min to system water only (Vehicle) under identical temperature, pH, salinity, oxygenation, and handling conditions. No additional substances were added to the control water.

The exposure timeline was as follows:

OXT 24 h groups received a single exposure (33.2 ng/mL for 15 min), with behavioral testing conducted 24 h after this exposure.

OXT 48 h groups received two identical exposures (33.2 ng/mL for 15 min), the first at baseline and the second 24 h later. Behavioral testing was performed 48 h after the first exposure (i.e., 24 h after the second exposure).

Vehicle groups underwent a single 15-minute exposure to drug-free system water and were behaviorally tested 24 h after exposure. No repeated Vehicle exposure was administered. No separate 48-hour Vehicle cohort was included; the single Vehicle condition tested at 24 h served as the baseline reference for both OXT dosing regimens within each phenotype. Importantly, only one Vehicle cohort was included in the study. Vehicle animals received a single 15-minute exposure at baseline and were behaviorally tested 24 h later. No Vehicle group was tested at 48 h, and no repeated vehicle exposure was performed. Thus, both OXT 24 h and OXT 48 h groups were compared to the same 24 h Vehicle baseline within each phenotype. This approach ensures that the factorial comparisons accurately reflect differences due to OXT dosing rather than the inclusion of additional vehicle exposures. Within the factorial model, the Vehicle condition represents the baseline reference group for each phenotype, against which both OXT 24 h (single exposure) and OXT 48 h (two exposures) groups were compared. Consequently, differences observed in the OXT 48 h condition reflect the combined effect of repeated dosing and an extended post-exposure interval relative to baseline vehicle conditions.

Accordingly, the factorial comparison comprised three dosing regimens (Vehicle, OXT 24 h – single exposure, OXT 48 h – two exposures). The second factorial factor represents an OXT dosing regimen rather than time alone, as the 48 h condition includes repeated exposure. To provide a clear overview of the experimental design, including OXT and Vehicle exposures and the timing of behavioral testing, the schedule for all zebrafish groups is illustrated in Figure 1.

Zebrafish were housed in groups of 10 per tank, with each tank corresponding to a specific phenotype × dosing condition, resulting in a total of six tanks. Exposure occurred collectively at the tank level; however, behavioral testing was conducted individually. Thus, the experimental unit for exposure was the tank, while the experimental unit for behavioral analysis was the individual fish, yielding *n* ═ 10 independent behavioral observations per group. Because a single tank was used per phenotype × dosing condition, tank-level random effects could not be formally modeled.

Sex distribution was slightly unbalanced across groups (A 24h: 6M/4F; A 48h: 5M/5F; N 24h: 5M/5F; N 48h: 6M/4F; CTR A: 5M/5F; CTR N: 6M/4F). Sex was not included as a covariate in the factorial model, as the study was not specifically powered to detect sex-dependent effects. The potential influence of sex is acknowledged as a methodological limitation.

### Behavioral tests

#### SI test

The apparatus consisted of a rectangular aquarium measuring 30 cm × 20 cm × 20 cm, filled with 6 L of water and divided into three compartments: a central neutral zone and two lateral stimulus zones, each containing either an albino conspecific or a non-albino (normal) conspecific. A camera was mounted above the aquarium to record fish activity throughout the test [36]. Fish were placed in the central zone for a 60-second adaptation period without exposure to lateral stimuli. Subsequently, partitions were removed, allowing for free exploration for 5 min. Behavioral patterns were analyzed with EthoVision XT 16 software (Noldus, The Netherlands), with measurements collected at 0.4-second intervals and averaged over 20-second bins (Figure 2).

**Figure 2.**
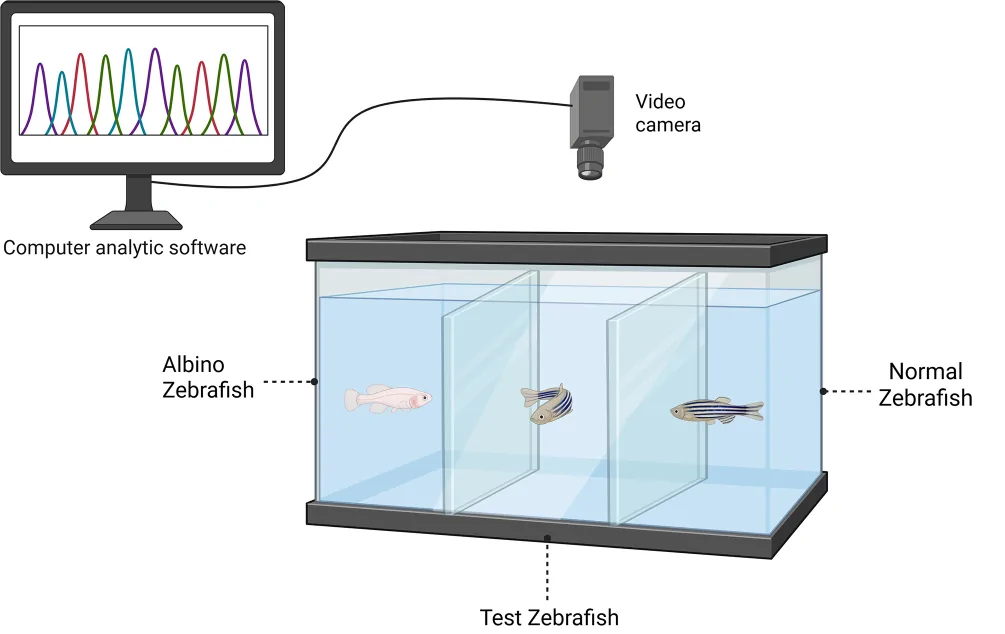
**SI test arena and video-tracking setup.** The rectangular aquarium (30 × 20 × 20 centimeters; 6 liters) was partitioned into three compartments: a central neutral zone for the test fish and two lateral stimulus zones containing an albino conspecific (left) and a non-albino conspecific (right). After a 60-second acclimation period in the neutral zone with partitions in place, partitions were removed and the test fish was allowed to explore freely for 5 minutes. Behavior was recorded by an overhead video camera and analyzed using EthoVision XT 16 (Noldus), with tracking sampled at 0.4-second intervals and averaged into 20-second bins. Schematic created with BioRender.com (accessed 11 October 2025). **Abbreviation:** SI, social interaction.

#### SPT

The experimental apparatus consisted of a T-shaped aquarium, with the central arm serving as a neutral starting zone and the two lateral arms functioning as stimulus compartments—one containing a conspecific and the other remaining empty. An overhead camera was mounted above the tank to continuously record fish activity throughout the test. Each individual fish was placed in the central arm of the maze for a 60-second adaptation period without visual access to the lateral compartments. In the SPT, the left arm of the tank always contained the conspecific stimulus, while the right arm remained empty (non-social arm). This fixed mapping ensures unambiguous interpretation of time spent in social vs non-social zones. All measurements were performed using EthoVision XT 16 (Noldus, The Netherlands) with the same tracking parameters as for the NTT. Following this step, the fish was allowed to freely explore the two lateral arms for 5 min, enabling assessment of preference between the social stimulus zone and the empty zone [37, 38] (Figure 3).

**Figure 3.**
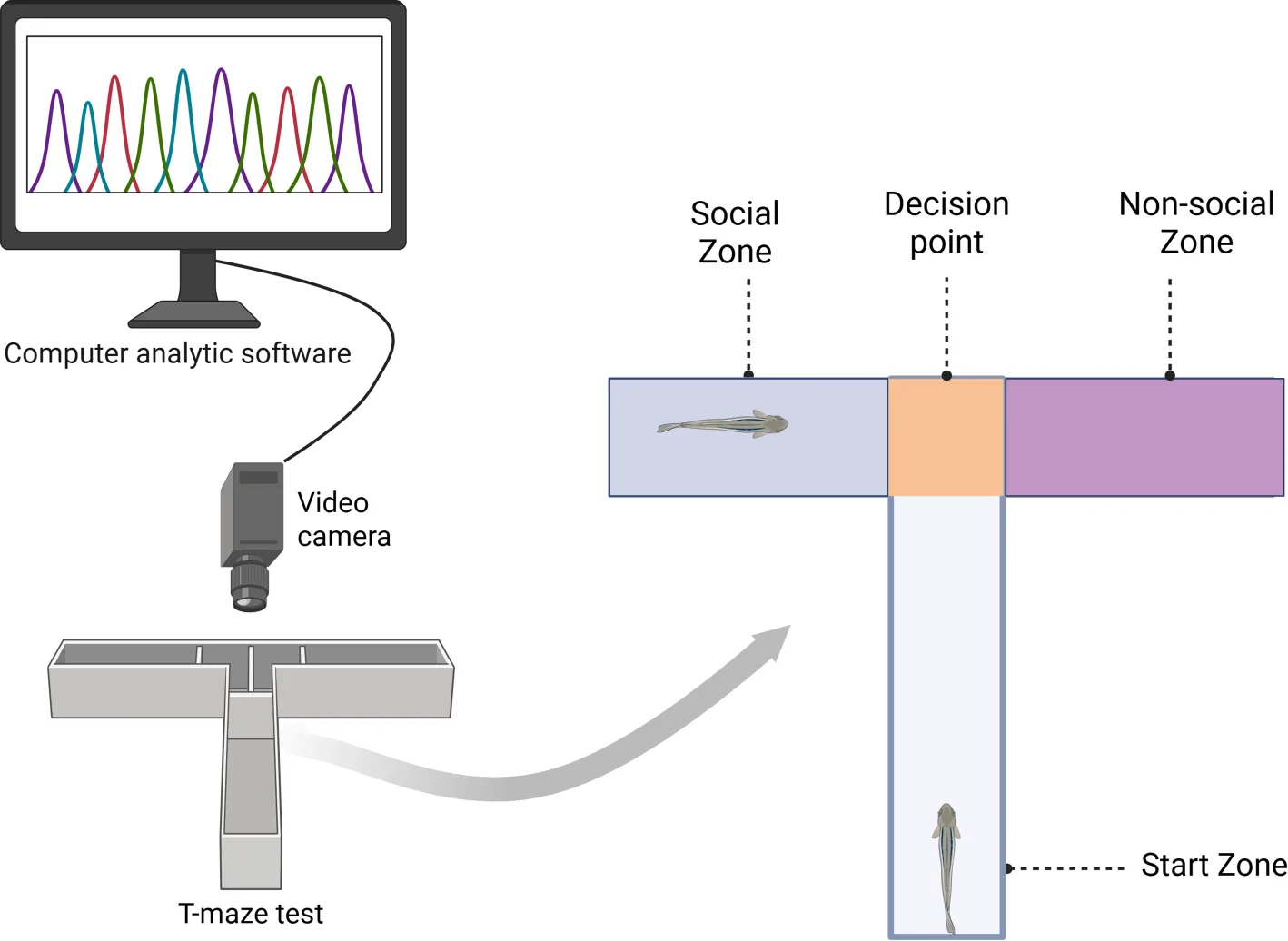
**SPT arena and zone definition.** The T-shaped tank comprised a central start arm and two lateral choice arms defining the social zone (left; conspecific stimulus present), decision point (junction), and non-social zone (right; empty). Each fish was placed in the start arm for 60 s acclimation without visual access to the lateral arms, after which barriers were removed and free exploration was recorded for 5 min using an overhead video camera and quantified in EthoVision XT 16 with tracking settings matched to the NTT. Schematic created with BioRender.com (accessed 11 October 2025). **Abbreviations:** NTT, novel tank test; SPT, social preference test.

#### NTT

To assess anxiety-like behavior and locomotor activity, the NTT was employed. Individual fish were gently transferred to a novel tank devoid of familiar environmental cues, and their behavior was recorded for 5–6 min. The lower part of the tank was operationally defined as the initial bottom zone, typically associated with anxiety-like behavior in zebrafish. For the NTT, mobility states of zebrafish were defined using EthoVision XT 16 (Noldus, The Netherlands) software, with the following velocity thresholds: immobile (freezing) < 0.5 cm/s, normal movement 0.5–3.0 cm/s, and high activity > 3.0 cm/s. Tracking was performed at 25 frames per second, using the default smoothing filter to ensure consistent automated scoring across all animals. These settings were uniformly applied to all fish to guarantee reproducibility of the behavioral classification. Increased exploration of the upper half was interpreted as reduced anxiety-like responses and/or behavioral adaptation to the novel configuration [39] (Figure 4).

**Figure 4.**
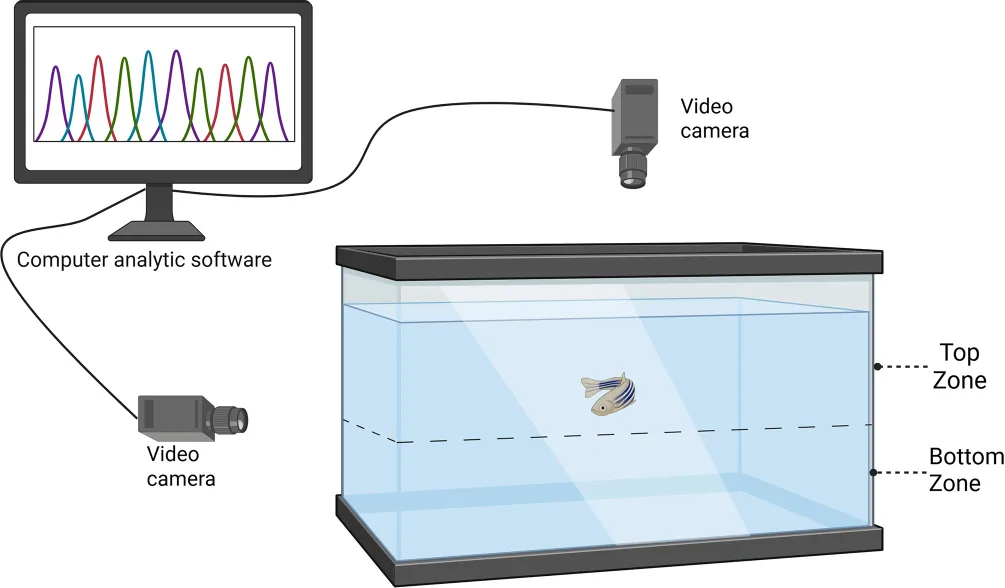
**NTT arena, zone definition, and automated tracking workflow.** Individual zebrafish were transferred to a novel tank and recorded for 5–6 min, with behavior captured by overhead and lateral video cameras for automated tracking. The tank was operationally divided into top and bottom zones, with the bottom zone representing the initial area typically associated with anxiety-like behavior. Locomotor states were quantified in EthoVision XT 16 using velocity thresholds: Immobile (<0.5 cm/s), mobile (0.5–3.0 cm/s), and highly mobile (>3.0 cm/s), with tracking performed at 25 fps. Increased exploration of the top zone was interpreted as reduced anxiety-like responses and/or adaptation to the novel environment. Schematic created with BioRender.com (accessed 11 October 2025). **Abbreviations:** fps, frames per second; NTT, novel tank test.

### Statistical analyses

All statistical analyses were performed using GraphPad Prism v9.1.0.221 (GraphPad Software, San Diego, CA, USA). Within the NTT parameters, measurements were quantified simultaneously from the top and side camera views and considered technical replicates of the same trajectory. To prevent pseudo-replication, values from the two camera views were averaged, generating a single value per subject per condition for subsequent analysis. This approach ensured that each fish contributed one independent value per condition.

Effect sizes were calculated for each term in the two-way ordinary analysis of variance (ANOVA) model, including Phenotype, Dosing regimen, and their interaction. Partial eta squared (ηp^2^), omega squared (ω^2^), and Cohen’s f were derived to quantify the magnitude of observed effects beyond statistical significance, in line with recommended practices for reporting effect sizes in behavioral research [40]. These additional measures complement F and *P* values by providing information on the proportion of variance explained and the relative strength of each factor and interaction, allowing for a more comprehensive interpretation of phenotypic and treatment effects on behavioral outcomes.

To directly test phenotype-dependent effects, data were analyzed using an ordinary two-way factorial ANOVA with phenotype (Normal vs. Albino) and dosing regimen (Vehicle; OXT single exposure assessed at 24h; OXT repeated exposure assessed at 48h). This approach allowed explicit evaluation of whether treatment effects differed between phenotypes. When significant main or interaction effects were detected, post-hoc comparisons were performed using Tukey’s multiple comparisons test to control the family-wise error rate. For post-hoc comparisons, adjusted mean differences and their 95% confidence intervals (95% CIs) were reported to provide an estimate of effect magnitude and precision. Parametric datasets were analyzed using one-way ANOVA with Tukey’s post hoc comparisons.

Given the relatively small sample size per experimental cell (*n* ═ 10) and the inclusion of proportion- and count-based behavioral endpoints, ANOVA assumptions were formally evaluated for all primary and secondary outcomes. Residual normality was assessed using Shapiro-Wilk tests and visual inspection of quantile-quantile plots (Q-Q plots), while homogeneity of variances across experimental cells was examined using Levene’s test. Although certain proportional and locomotor endpoints exhibited deviations from normality, homogeneity of variance was consistently confirmed across all factorial conditions. Considering the balanced 2 × 3 design, ANOVA robustness to moderate deviations from normality was deemed acceptable. To further address potential assumption violations, all factorial models were additionally confirmed using heteroscedasticity-consistent (HC3) robust standard errors. Robust estimation yielded identical inferential conclusions for all main and interaction effects.

All data are presented as mean ± standard deviation (SD), with *P <* 0.05 considered statistically significant. Primary social endpoints (social interaction and social preference metrics) were controlled for multiplicity using the Benjamini-Hochberg False Discovery Rate (FDR) procedure (*Q* ═ 0.05). Adjusted q-values are reported alongside the corresponding primary endpoints.

Sex was not included as a covariate in the final factorial model, as the study was not specifically powered to detect sex-dependent effects. The potential influence of sex is acknowledged as a limitation.

## Results

A comprehensive behavioral profiling battery was conducted, encompassing locomotor, spatial, directional, and mobility-related parameters. In accordance with the predefined analytical framework, behavioral outcomes were systematically categorized into social-spectrum indices (primary endpoints) and locomotor features (secondary endpoints).

Spatial parameters such as time spent in predefined zones and distance to a target zone were conceptualized as social-spectrum indices. These variables capture stimulus-directed spatial allocation and proximity-based approach behavior, serving as direct indicators of social affiliation, stimulus preference, and decision-processing dynamics. Unlike global locomotor output measures, zone occupancy and distance-to-zone metrics reflect goal-directed positioning relative to behaviorally relevant stimuli. Accordingly, they were designated as primary social endpoints within the analytical framework, as they more specifically index social approach behavior rather than generalized motor activation.

Across all behavioral paradigms, locomotor features were operationally defined as quantitative indices of global motor output and locomotor state distribution. These included swimming distance, velocity, mobility-state allocation, and directional turning behavior (clockwise and counterclockwise rotations). Collectively, these parameters reflect overall motor activation, arousal state, and movement dynamics rather than stimulus-oriented spatial allocation.

Full estimates of mean differences with 95% CIs for all pairwise contrasts are provided in Supplementary Table S1.

### Behavioral response of zebrafish in the SPT

#### Social phenotype features

The primary outcome measures were predefined spatial indices that reflect social affiliation and decision-making behavior. These indices included: (I) time spent in relevant compartments, indicating social preference and engagement in social choice processing; and (II) mean distance to a predefined zone, reflecting directional spatial bias. These variables were chosen because they directly quantify phenotype-dependent modulation of social approach behavior and spatial preference following OXT administration.

For cumulative in-zone duration (%) in the left stimulus arm (Figure 5), two-way ordinary ANOVA revealed no significant interaction, F(2,54) ═ 1.385, *P ═* 0.259, ηp^2^ ═ 0.048, ω^2^ ═ 0.012, *f* ═ 0.226. The main effect of phenotype was not significant, F(1,54) ═ 0.034, *P ═* 0.854, ηp^2^ < 0.001, ω^2^ ═ 0.000, *f* ═ 0.025, indicating negligible variance attributable to pigmentation status. A trend toward a main effect of dosing was observed, F(2,54) ═ 2.787, *P ═* 0.070, ηp^2^ ═ 0.093, ω^2^ ═ 0.056, *f* ═ 0.321, suggesting a modest influence of OXT dosing on social arm occupancy. Tukey’s HSD post hoc comparisons revealed that, within the non-albino group, OXT at 48 h differed significantly from Vehicle (adjusted *P ═* 0.036), whereas no significant pairwise differences were detected within the albino phenotype.

**Figure 5.**
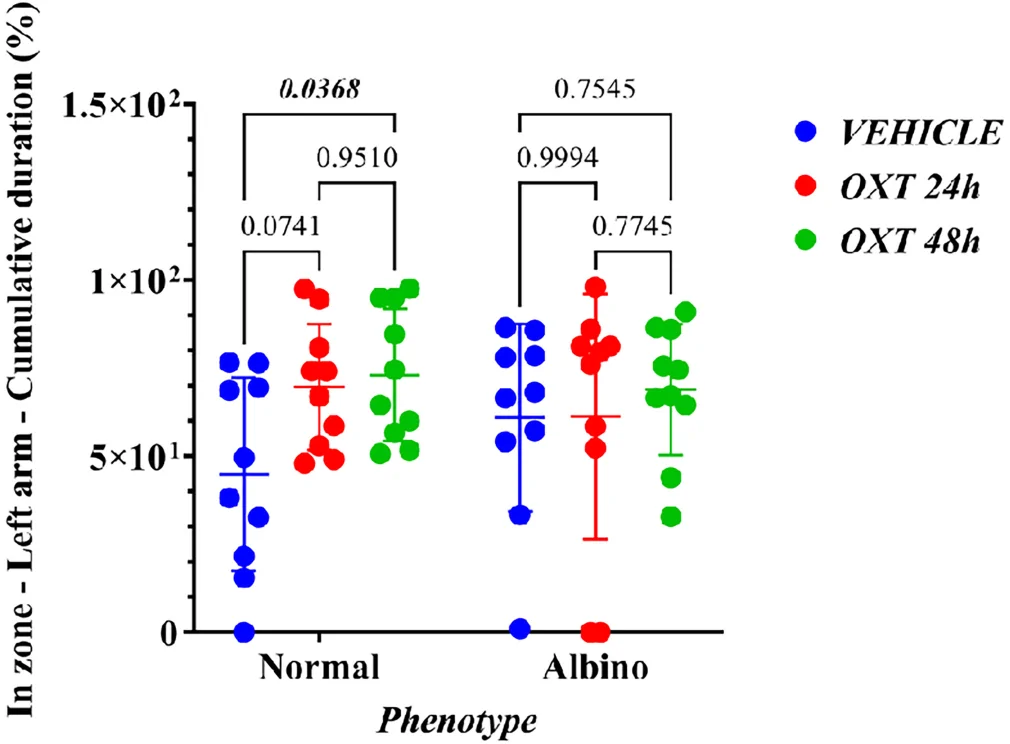
**Cumulative time in the left (social) arm in the SPT.** Percent time spent in the left arm (conspecific stimulus) by non-albino and albino zebrafish following Vehicle, OXT 24-hour (single exposure), or OXT 48-hour (two exposures) regimens. Each dot represents one fish (*n* ═ 10 per group); horizontal lines and whiskers indicate mean ± standard deviation. Group differences were evaluated by two-way ordinary factorial analysis of variance (Phenotype × Dosing regimen) followed by Tukey’s multiple-comparisons post hoc test; adjusted *P*-values for within-phenotype contrasts are shown above brackets. **Abbreviations:** OXT, oxytocin; SPT, social preference test.

Similarly, for cumulative time spent (%) in the decision zone (Figure 6), two-way ANOVA demonstrated no significant interaction, F(2,54) ═ 0.046, *P ═* 0.954, ηp^2^ ═ 0.002, ω^2^ ═ 0.000, *f* ═ 0.042. The main effect of phenotype was not significant, F(1,54) ═ 0.337, *P ═* 0.563, ηp^2^ ═ 0.006, ω^2^ ═ 0.000, *f* ═ 0.079. In contrast, a highly significant main effect of dosing was observed, F(2,54) ═ 11.41, *P <* 0.0001, ηp^2^ ═ 0.297, ω^2^ ═ 0.266, *f* ═ 0.650, indicating a large treatment-related effect independent of phenotype. These findings demonstrate that OXT dosing robustly modulated occupancy of the central decision zone regardless of pigmentation status. Tukey’s HSD post hoc comparisons indicated that, within the non-albino group, OXT at 24 and 48 h differed significantly from Vehicle (adjusted *P ═* 0.018/*P ═* 0.005), while a significant pairwise difference was detected within the albino phenotype at 48 h post-OXT administration (adjusted *P ═* 0.008). This primary social endpoint remained significant after FDR correction (*q* ═ 0.0003).

**Figure 6.**
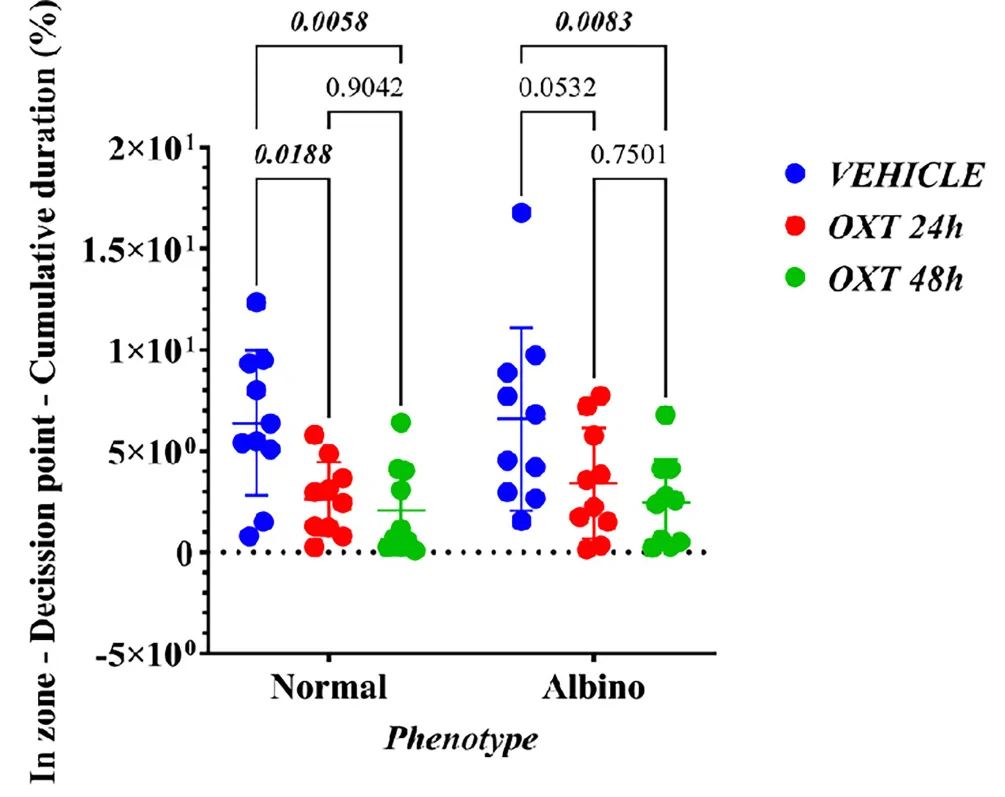
**Cumulative time in the decision zone in the SPT.** Percent time spent in the central decision point by non-albino and albino zebrafish following Vehicle, OXT 24-hour (single exposure), or OXT 48-hour (two exposures) regimens. Each dot represents one fish (*n* ═ 10 per group); horizontal lines and whiskers indicate mean ± standard deviation. Group differences were evaluated by two-way ordinary factorial analysis of variance (Phenotype × Dosing regimen) followed by Tukey’s multiple-comparisons post hoc test; adjusted *P*-values for within-phenotype contrasts are shown above brackets. This endpoint remained significant after FDR correction (q = 0.0003). **Abbreviations:** FDR, false discovery rate; OXT, oxytocin; SPT, social preference test.

For mean distance (cm) to the non-social arm (Figure 7), two-way ordinary ANOVA revealed no significant interaction, F(2,54) ═ 0.314, *P ═* 0.731, ηp^2^ ═ 0.011, ω^2^ ═ 0.000, *f* ═ 0.107. The main effect of phenotype was also not significant, F(1,54) ═ 0.114, *P ═* 0.736, ηp^2^ ═ 0.002, ω^2^ ═ 0.000, *f* ═ 0.045, indicating negligible variance attributable to pigmentation status. In contrast, a significant main effect of dosing was observed, F(2,54) ═ 5.352, *P ═* 0.007, ηp^2^ ═ 0.165, ω^2^ ═ 0.131, *f* ═ 0.445, reflecting a moderate-to-large effect size. These findings indicate that distance to the non-social arm was significantly modulated by treatment dosing, independent of phenotype, with minimal contribution from interaction or pigmentation status. Tukey’s HSD post hoc comparisons revealed that, within the non-albino group, OXT at 48 h differed significantly from Vehicle (adjusted *P ═* 0.036), whereas no significant differences were observed within the experimental group. This effect remained significant after FDR correction (*q* ═ 0.0097).

**Figure 7.**
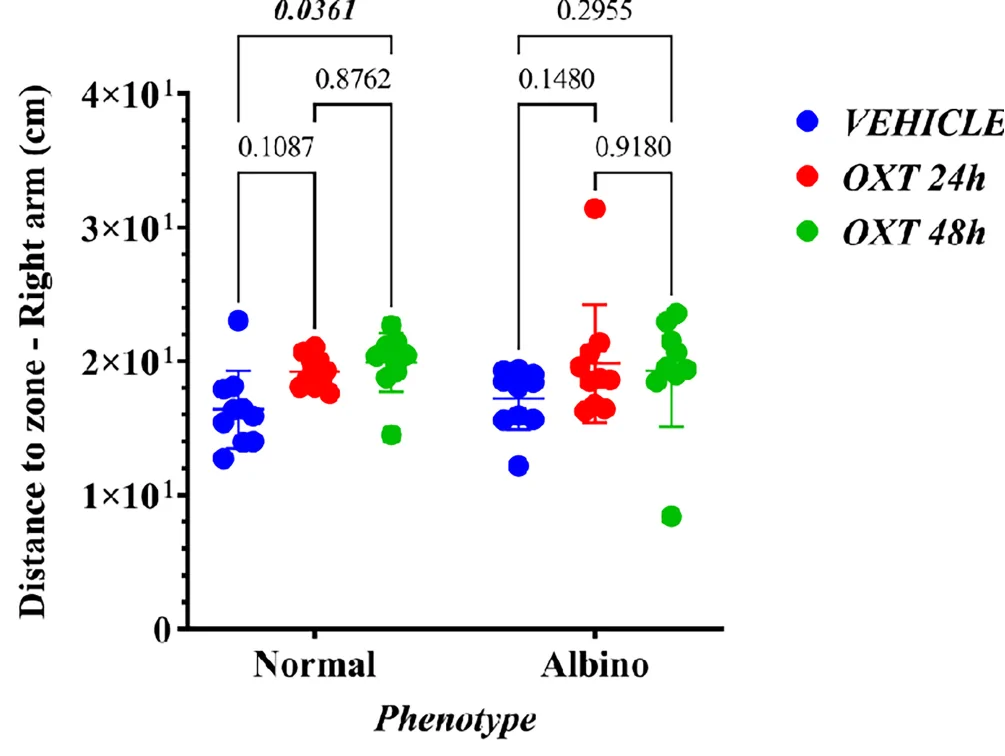
**Mean distance to the non-social arm in the SPT.** Mean distance (cm) from the test fish to the right (non-social) arm in non-albino and albino zebrafish following Vehicle, OXT 24-hour (single exposure), or OXT 48-hour (two exposures) regimens. Each dot represents one fish (*n* ═ 10 per group); horizontal lines and whiskers indicate mean ± standard deviation. Group differences were evaluated by two-way ordinary factorial analysis of variance (Phenotype × Dosing regimen) followed by Tukey’s multiple-comparisons post hoc test; adjusted *P*-values for within-phenotype contrasts are shown above brackets. This endpoint remained significant after FDR correction (q = 0.0097). **Abbreviations:** FDR, false discovery rate; OXT, oxytocin; SPT, social preference test.

#### Locomotor phenotype features

Adjacent locomotor and directional parameters, including swimming distance (cm), velocity (cm/s), and counterclockwise rotation (frequency), were analyzed as control indices of global motor output.

Although significant differences were observed, these were interpreted as modulation of global locomotor dynamics rather than direct alterations of social preference. Importantly, the dissociation between spatial-social effects and locomotor modulation supports the interpretation that OXT influenced social decision-making processes beyond simple motor activation changes.

Swimming distance (cm) (Figure 8), analyzed as an index of global locomotor output, showed no significant interaction between phenotype and dosing, F(2,54) ═ 0.918, *P ═* 0.405, ηp^2^ ═ 0.033, ω^2^ ═ 0.000, *f* ═ 0.184, indicating that treatment effects did not differ between normal and albino fish. The main effect of phenotype was not significant, F(1,54) ═ 1.927, *P ═* 0.170, ηp^2^ ═ 0.034, ω^2^ ═ 0.014, *f* ═ 0.189, suggesting comparable overall locomotor output between pigmentation groups. However, a significant main effect of OXT dosing was observed, F(2,54) ═ 3.407, *P ═* 0.040, ηp^2^ ═ 0.112, ω^2^ ═ 0.073, *f* ═ 0.355, indicating dosing-dependent modulation of swimming activity independent of phenotype. Tukey’s HSD post hoc analysis revealed a significant difference between Vehicle and OXT at 48 h within the normal phenotype (adjusted *P ═* 0.048), while no significant pairwise contrasts were detected in albino fish.

**Figure 8.**
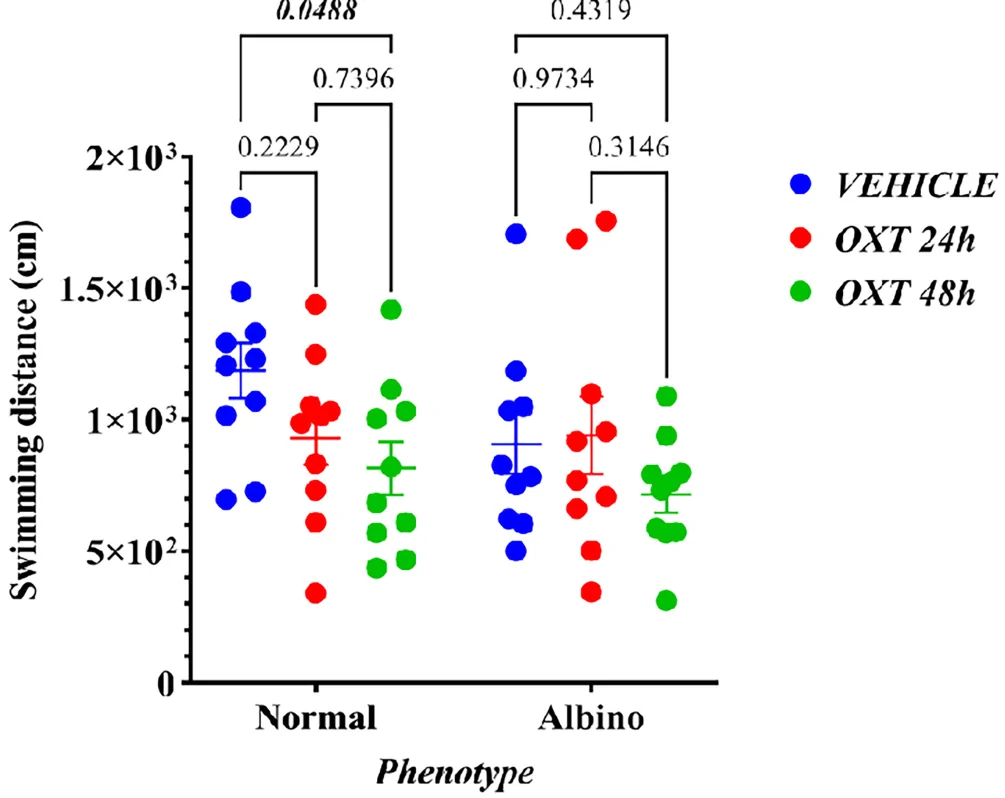
**Swimming distance in the SPT.** Total swimming distance (cm), used as an index of global locomotor output during the SPT, in non-albino and albino zebrafish following Vehicle, OXT 24-hour (single exposure), or OXT 48-hour (two exposures) regimens. Each dot represents one fish (*n* ═ 10 per group); horizontal lines and whiskers indicate mean ± standard deviation. Group differences were evaluated by two-way ordinary factorial analysis of variance (Phenotype × Dosing regimen) followed by Tukey’s multiple-comparisons post hoc test; adjusted *P*-values for within-phenotype contrasts are shown above brackets. **Abbreviations:** OXT, oxytocin; SPT, social preference test.

These findings indicate that OXT exposure modestly influenced global locomotor activity in a dosing-dependent manner, with limited phenotype specificity. Importantly, the absence of interaction effects supports the interpretation that spatial-social outcomes observed in the primary endpoints cannot be solely attributed to differential locomotor activation.

For the velocity (cm/s) parameter (Figure 9), two-way ordinary ANOVA revealed no significant interaction; F(2,54) ═ 0.928, *P ═* 0.401, ηp^2^ ═ 0.033, ω^2^ ═ 0.000, *f* ═ 0.186, indicating that treatment-related changes in velocity did not differ between phenotypes. The main effect of phenotype was also not significant, F(1,54) ═ 1.901, *P ═* 0.173, ηp^2^ ═ 0.034, ω^2^ ═ 0.016, *f* ═ 0.188, suggesting comparable overall swimming speeds between normal and albino zebrafish. In contrast, a significant main effect of OXT dosing was observed, F(2,54) ═ 3.390, *P ═* 0.041, ηp^2^ ═ 0.112, ω^2^ ═ 0.077, *f* ═ 0.354, indicating a modest OXT-dependent modulation of locomotor velocity independent of pigmentation status.

**Figure 9.**
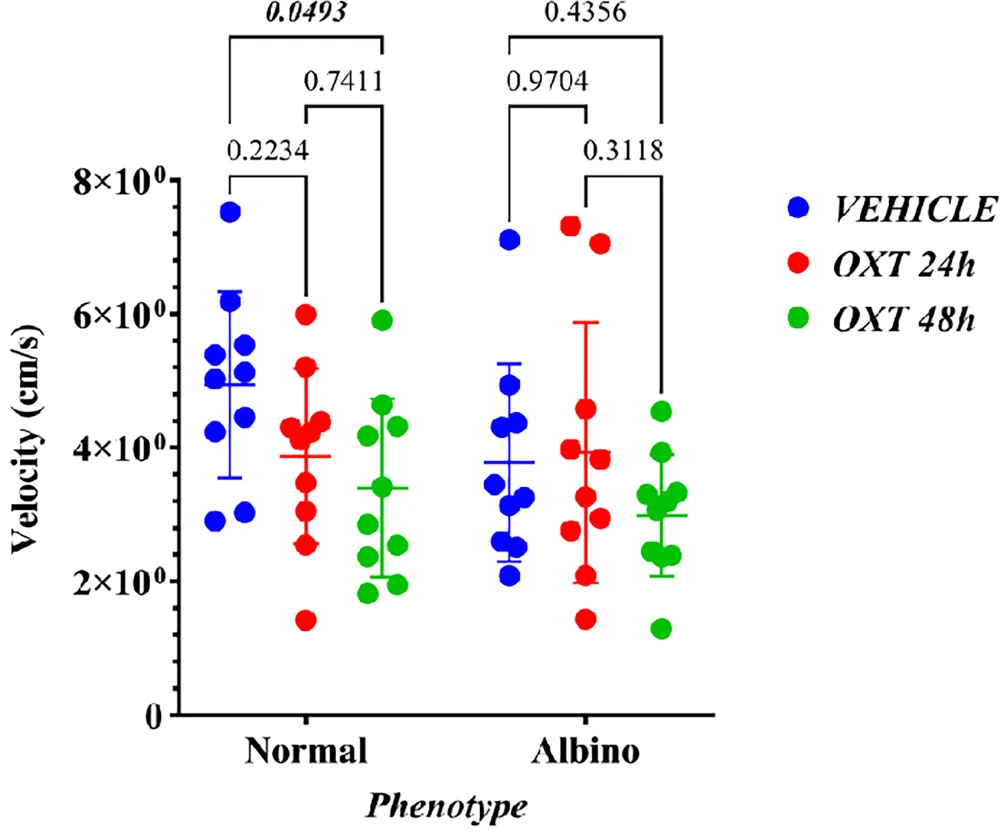
**Swimming velocity in the SPT.** Mean swimming velocity (cm/s), used as an index of global locomotor output during the SPT, in non-albino and albino zebrafish following Vehicle, OXT 24-hour (single exposure), or OXT 48-hour (two exposures) regimens. Each dot represents one fish (*n* ═ 10 per group); horizontal lines and whiskers indicate mean ± standard deviation. Group differences were evaluated by two-way ordinary factorial analysis of variance (Phenotype × Dosing regimen) followed by Tukey’s multiple-comparisons post hoc test; adjusted *P*-values for within-phenotype contrasts are shown above brackets. **Abbreviations:** OXT, oxytocin; SPT, social preference test.

Tukey’s HSD post hoc comparisons identified a significant contrast exclusively within the normal phenotype (Vehicle vs. OXT at 48 h, adjusted *P ═* 0.049), whereas no significant pairwise differences were detected within the albino group (all adjusted *P* > 0.05).

For counterclockwise rotations (frequency) (Figure 10), two-way ordinary ANOVA revealed no significant interaction, F(2,54) ═ 0.323, *P ═* 0.725, ηp^2^ ═ 0.012, ω^2^ ═ 0.000, *f* ═ 0.109, indicating that dosing-dependent modulation did not differ between normal and albino fish. The main effect of phenotype was not significant, F(1,54) ═ 0.006, *P ═* 0.936, ηp^2^ < 0.001, ω^2^ ═ 0.000, *f* ═ 0.011, demonstrating comparable overall turning frequency between pigmentation groups.

**Figure 10.**
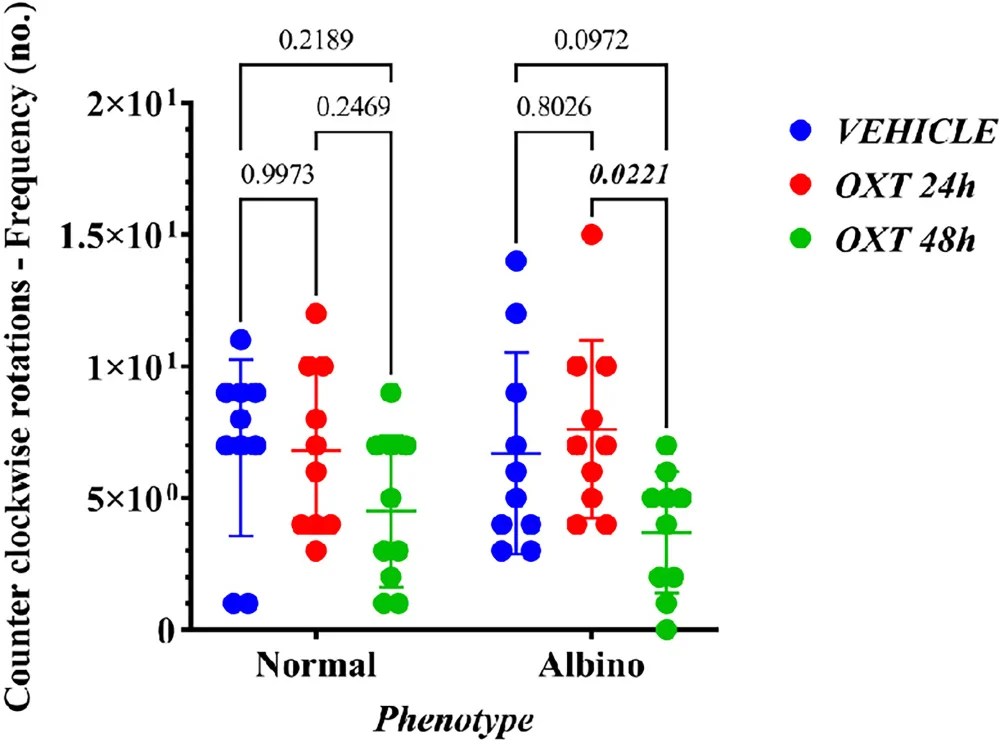
**Counterclockwise rotations in the SPT.** Frequency of counterclockwise rotations (no.), used as an index of directional locomotor dynamics during the SPT, in non-albino and albino zebrafish following Vehicle, OXT 24-hour (single exposure), or OXT 48-hour (two exposures) regimens. Each dot represents one fish (*n* ═ 10 per group); horizontal lines and whiskers indicate mean ± standard deviation. Group differences were evaluated by two-way ordinary factorial analysis of variance (Phenotype × Dosing regimen) followed by Tukey’s multiple-comparisons post hoc test; adjusted *P*-values for within-phenotype contrasts are shown above brackets. **Abbreviations:** OXT, oxytocin; SPT, social preference test.

In contrast, a significant main effect of OXT dosing was observed, F(2,54) ═ 5.628, *P ═* 0.006, ηp^2^ ═ 0.172, ω^2^ ═ 0.138, *f* ═ 0.457, indicating moderate-to-large dosing-dependent modulation of rotational behavior independent of phenotype.

Tukey’s HSD post hoc analysis showed no significant pairwise differences within the normal phenotype (all adjusted *P* > 0.05). However, within the albino group, a significant increase in counterclockwise rotations was detected between OXT at 24 h and OXT at 48 h (adjusted *P ═* 0.022), while comparisons between Vehicle vs. OXT at 24 h/OXT at 48 h were not significant.

Collectively, these findings indicate that OXT exposure induced a dosing-dependent modulation of rotational motor dynamics, with a delayed effect observed selectively in the albino phenotype. Importantly, because rotational frequency represents a directional locomotor index rather than a spatial-social allocation measure, these changes were interpreted as modulation of motor patterning rather than direct alterations of social preference behavior.

### Behavioral response of zebrafish in the SI test

#### Social phenotype features

Within the SI test, a comprehensive behavioral profiling battery was applied, encompassing multiple branched behavioral features. Consistent with the conceptual framework established above, the inferential focus remained on spatial indices reflecting social affiliation and decision-making behavior. Accordingly, time spent in stimulus-associated compartments was considered the primary social outcome measure. These spatial metrics directly quantify social approach tendencies and relative stimulus preference, independent of absolute locomotor output.

Cumulative duration (%) in the stimulus-associated zone was analyzed as a primary spatial endpoint reflecting social affiliation (data not shown). Two-way ordinary ANOVA showed no significant interaction, F(2,54) ═ 0.599, *P ═* 0.552, ηp^2^ ═ 0.022, ω^2^ ═ 0.000, *f* ═ 0.149, indicating that the effect of OXT dosing regimen did not differ between phenotypes. A significant main effect of phenotype was observed, F(1,54) ═ 7.298, *P ═* 0.009, ηp^2^ ═ 0.119, ω^2^ ═ 0.097, *f* ═ 0.368, suggesting an overall difference in stimulus-zone occupancy between albino and normal fish independent of OXT dosing regimen. The main effect of OXT dosing was not significant, F(2,54) ═ 0.742, *P ═* 0.480, ηp^2^ ═ 0.027, ω^2^ ═ 0.000, *f* ═ 0.166. The phenotype effect on this primary social endpoint remained significant after FDR correction (*q* ═ 0.0097).

Although the global phenotype effect was significant, Tukey’s HSD post hoc comparisons did not identify significant pairwise differences within phenotypes across timepoints (all adjusted *P* > 0.05), consistent with a broad phenotypic shift rather than discrete condition-specific contrasts.

#### Locomotor phenotype features

From the extensive array of behavioral parameters analyzed (Table S2), the locomotor indices exhibiting significant global effects are presented in the table below. These analyses aimed to ensure a comprehensive behavioral characterization and to assess whether global motor modulation could influence the interpretation of predefined primary social-spatial endpoints.

### Behavioral response of zebrafish in the NTT

In the NTT, significant pairwise differences were identified exclusively for locomotor-related parameters, particularly in the cumulative duration among mobility states (%).

The cumulative duration in the highly mobile state (%) (Figure 11) demonstrated a significant interaction, F(2,54) ═ 6.889, *P ═* 0.002, ηp^2^ ═ 0.203, ω^2^ ═ 0.166, *f* ═ 0.505, indicating a large interaction effect. Significant main effects were also observed for phenotype, F(1,54) ═ 13.07, *P <* 0.001, ηp^2^ ═ 0.195, ω^2^ ═ 0.159, *f* ═ 0.492, and dosing, F(2,54) ═ 11.48, *P <* 0.001, ηp^2^ ═ 0.298, ω^2^ ═ 0.263, *f* ═ 0.652, demonstrating substantial treatment-related modulation of locomotor activity.

**Figure 11.**
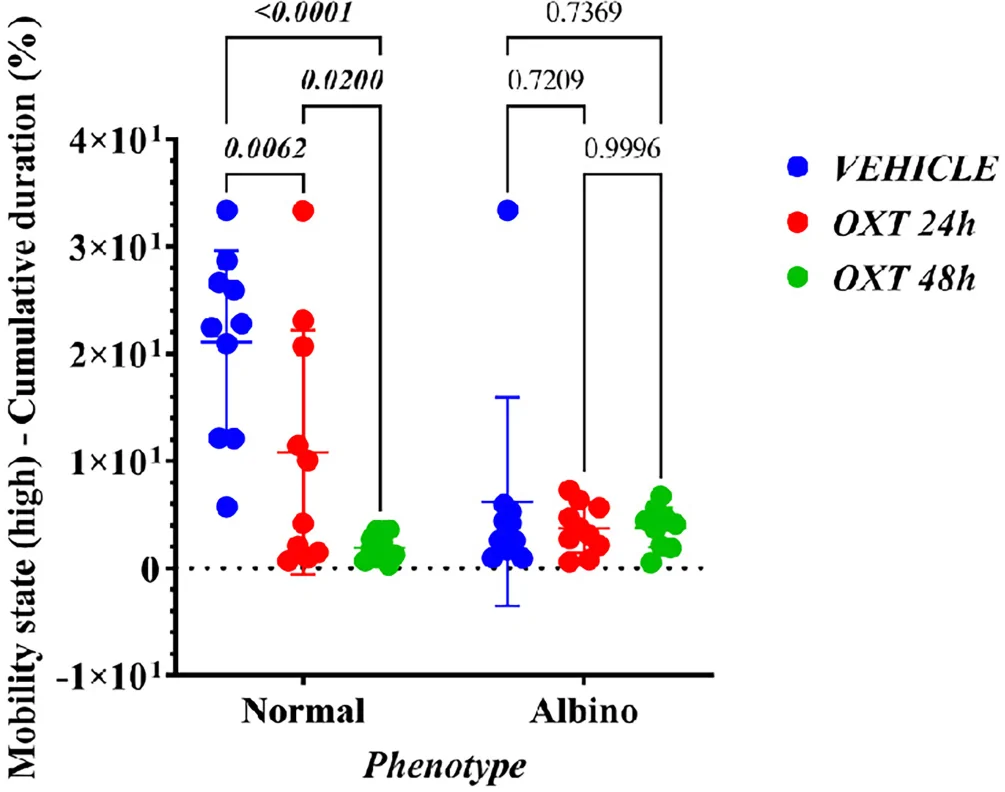
**Highly mobile state duration in the NTT.** Cumulative duration spent in the highly mobile state (%), quantified during the NTT in non-albino and albino zebrafish following Vehicle, OXT 24-hour (single exposure), or OXT 48-hour (two exposures) regimens. Each dot represents one fish (*n* ═ 10 per group); horizontal lines and whiskers indicate mean ± standard deviation. Group differences were evaluated by two-way ordinary factorial analysis of variance (Phenotype × Dosing regimen) followed by Tukey’s multiple-comparisons post hoc test; adjusted *P*-values for within-phenotype contrasts are shown above brackets. **Abbreviations:** NTT, novel tank test; OXT, oxytocin.

Tukey’s HSD post hoc analysis revealed significant pairwise differences solely within the Normal phenotype (Vehicle vs. OXT 24h, *P ═* 0.006; Vehicle vs. OXT 48h, *P <* 0.001; OXT 24 h vs. OXT 48h, *P ═* 0.020), while no significant pairwise differences were detected within the Albino group. These findings suggest that OXT exposure markedly modulated high-activity locomotor states in the NTT in a phenotype-dependent manner, with pronounced effects observed in Normal but not Albino zebrafish.

Similarly, for the cumulative duration of the immobile state (%) (Figure 12), a significant interaction was observed, F(2,54) ═ 4.514, *P ═* 0.015, ηp^2^ ═ 0.143, ω^2^ ═ 0.109, *f* ═ 0.408, alongside a significant main effect of OXT dosing, F(2,54) ═ 5.674, *P ═* 0.005, ηp^2^ ═ 0.174, ω^2^ ═ 0.138, *f* ═ 0.459. The main effect of phenotype did not reach statistical significance, F(1,54) ═ 3.598, *P ═* 0.063, ηp^2^ ═ 0.063, ω^2^ ═ 0.032, *f* ═ 0.260. Pairwise comparisons again indicated significant differences within the Normal phenotype only, specifically between Vehicle and OXT after 24/48h (adjusted *P ═* 0.045/*P <* 0.001) with no significant treatment contrasts in Albino fish.

**Figure 12.**
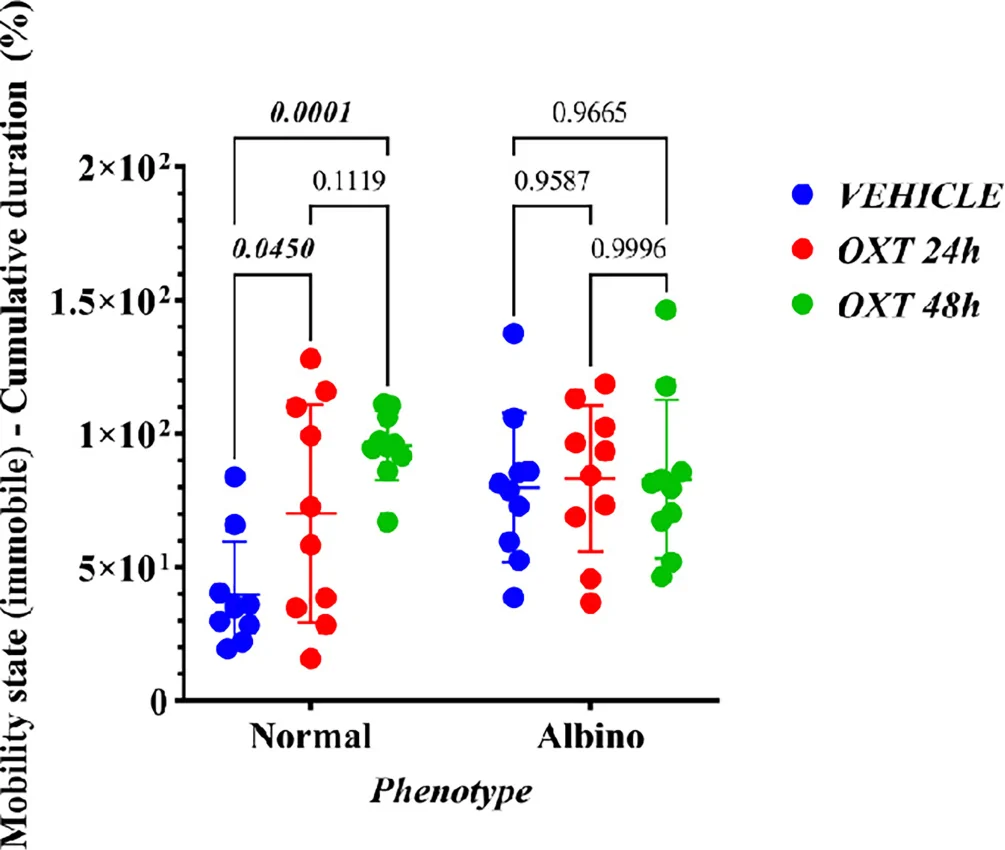
**Immobile state duration in the NTT.** Cumulative duration spent in the immobile state (%), quantified during the NTT in non-albino and albino zebrafish following Vehicle, OXT 24-hour (single exposure), or OXT 48-hour (two exposures) regimens. Each dot represents one fish (*n* ═ 10 per group); horizontal lines and whiskers indicate mean ± standard deviation. Group differences were evaluated by two-way ordinary factorial analysis of variance (Phenotype × Dosing regimen) followed by Tukey’s multiple-comparisons post hoc test; adjusted *P*-values for within-phenotype contrasts are shown above brackets. **Abbreviations:** NTT, novel tank test; OXT, oxytocin.

Collectively, these findings indicate that OXT exposure in the NTT primarily modulated locomotor state distribution, with pronounced dosing-related effects and stronger responsiveness in the Normal phenotype.

In the NTT, additional locomotor parameters exhibited significant global effects beyond mobility-state distribution. For clockwise rotations (count), data indicated a moderate phenotypic difference in directional locomotor behavior independent of the dosing regimen. No significant interaction or main effect of dosing was observed. Similarly, the cumulative duration of the active state demonstrated a significant global effect, reflecting modulation of overall locomotor activation patterns rather than vertical zone allocation. These results indicate that OXT exposure influenced general motor output dynamics within the NTT, while not consistently altering vertical spatial exploration parameters. The complete statistical outputs for clockwise rotations and activity-state variables are presented in Table S3.

All primary social endpoints remained significant after Benjamini-Hochberg FDR correction (*Q* ═ 0.05), with adjusted q values ranging from 0.0003 for decision-point occupancy (%) in the SPT to 0.0097 for distance to the right arm (cm) in the SPT and in-zone time (%) in the SI test, confirming the robustness of the primary social effects under multiplicity control.

Residual diagnostics indicated deviations from normality for certain proportional and locomotor endpoints; however, homogeneity of variance was confirmed across experimental cells. HC3 robust analyses yielded identical statistical conclusions for all primary and secondary outcomes, confirming that the reported effects were not driven by assumption violations.

## Discussion

The present study investigated whether OXT exerts phenotype-dependent behavioral effects in adult zebrafish using a fully factorial 2 × 3 design (phenotype × treatment/dosing). By analytically distinguishing predefined primary social-spatial endpoints from secondary locomotor parameters, the findings provide a structured evaluation of how OXT modulates social allocation and anxiety-related motor dynamics across contexts.

### Influence of OXT dosing regimen on social allocation

Across structured social paradigms, including the SPT and SI test, OXT effects were predominantly characterized by significant main effects of the dosing regimen, while phenotype × treatment interactions were limited for primary social endpoints.

Previous research has demonstrated that OXT signaling regulates temporal aspects of social behavior in zebrafish. Disruption of OXT receptors (Oxtr/Oxtrl) alters the development and maintenance of social preference and social recognition without uniformly affecting baseline social motivation. Moreover, OXT receptor signaling has been shown to modulate novelty recognition rather than social preference per se [41], and developmental OXT signaling influences social information processing [42].

Recent data also indicate that OXT can enhance time-dependent behavioral responses in zebrafish [43]. Taken together, these findings support the interpretation that OXT modulates the dynamics and processing of social decisions over time rather than merely increasing or decreasing sociality.

Importantly, in the present study, robust phenotype × treatment interactions were not observed in the main social allocation parameters. This suggests that pigmentation phenotype did not fundamentally alter OXT-driven social allocation under the current experimental conditions.

### Phenotype-specific modulation in anxiety-related locomotor dynamics

In contrast to structured social measures, phenotype-dependent modulation was more pronounced in locomotor parameters within the NTT. The duration of high mobility and immobility demonstrated significant phenotype × treatment interactions, indicating differential sensitivity of anxiety-related motor states to OXT exposure.

The NTT serves as a well-established measure of anxiety-like behavior and stress adaptation in zebrafish. OXT has been implicated in stress regulation and social modulation across vertebrates, with recent evidence suggesting that social cues and OXT receptors influence stress recovery dynamics in zebrafish [44]. Additionally, OXT signaling has been proposed as a biomarker for environmental stressors [45].

At the circuit level, zebrafish OXT neurons can drive defensive locomotor responses through brainstem premotor targets [46], providing a mechanistic framework for understanding OXT-related modulation of high-activity states in novel environments. In the present study, non-albino fish exhibited more pronounced OXT-related shifts in locomotor state distribution across the OXT dosing regimen, while albino fish displayed attenuated or less differentiated responses [47].

These findings suggest that phenotypic background influences the sensitivity of anxiety-related locomotor regulation to OXT, particularly in anxiogenic contexts.

### Dissociation between social allocation and general motor output

A notable strength of this study is the separation of social-spatial allocation metrics from overall locomotor indices. Several locomotor parameters showed treatment-related changes without corresponding shifts in social decision-zone occupancy. This dissociation indicates that OXT-induced modulation of social allocation cannot be solely attributed to generalized changes in motor activity.

Previous behavioral frameworks emphasize the importance of distinguishing anxiety-related locomotor changes from stimulus-directed social preferences. By employing a factorial design and reporting interaction terms, the present data support the partially independent modulation of social and locomotor domains by OXT.

### Potential role of pigmentation-related sensory processing

Albino zebrafish lack normal ocular pigmentation due to alterations in melanin synthesis pathways. Reduced ocular pigmentation may influence visual processing and stimulus discrimination. Indeed, perceptual mechanisms play a crucial role in social affiliation in zebrafish [48], and phenotype-related differences in stimulus discrimination have been reported between albino and non-albino models [49].

Although visual function was not directly assessed in the present study, altered sensory processing could contribute to phenotype-related differences in locomotor stress adaptation or subtle spatial allocation patterns. Future studies integrating visual assays and receptor-expression analyses will clarify the mechanistic basis of these effects.

### Strengths and limitations of the study

The present study possesses several methodological strengths. First, a fully factorial 2 × 3 experimental design facilitated explicit testing of phenotype × treatment interactions, enabling direct assessment of phenotype-dependent OXT effects. Second, individual behavioral testing minimized measurement noise and ensured independence in outcome assessment. Third, effect sizes (ηp^2^, ω^2^, Cohen’s f) were systematically reported alongside F and *P* values, with robustness analyses using HC3 estimation confirming the stability of all inferential conclusions. Collectively, these features enhance the reliability and interpretability of the reported behavioral effects.

However, several limitations warrant acknowledgment. Although both male and female zebrafish were included in all experimental groups, the sex distribution was not fully balanced across conditions, and sex was not incorporated as an independent factor in the factorial model. The study was not specifically powered to detect sex-dependent behavioral differences; hence, subtle sex-related modulation of OXT responsiveness cannot be excluded and should be addressed in future investigations using balanced and adequately powered designs.

Additionally, the fish were exposed collectively within one tank per treatment condition. While behavioral testing was conducted individually, this exposure design does not permit formal modeling of tank-level random effects; thus, potential clustering at the tank level cannot be fully ruled out. Future studies including multiple tanks per treatment condition would allow for hierarchical statistical modeling and further enhance inferential precision.

Moreover, the albino phenotype was identified visually rather than through genetic verification, which could introduce variability in classification and limit the generalizability of findings. The lack of ocular pigmentation in albino fish may also affect visual perception, potentially influencing social and locomotor behaviors. Future research should incorporate genetic confirmation of phenotype and consider assessing visual function or receptor expression to elucidate underlying mechanisms. Furthermore, although locomotor activity was measured, vertical positioning metrics in the NTT were not analyzed as primary endpoints, suggesting that conclusions regarding exploration-related or anxiety-like behaviors should be drawn with caution.

## Conclusion

In conclusion, OXT exerts context- and dosing-regimen-dependent behavioral effects in zebrafish. Social allocation parameters in structured social paradigms were primarily modulated by OXT dosing regimen and exhibited a degree of independence from phenotype, whereas locomotor state dynamics in the NTT displayed clear phenotype-specific sensitivity, with stronger mobility-state modulation in the non-albino strain. These findings indicate that OXT differentially influences social decision-processing and anxiety-related locomotor regulation across behavioral contexts. Overall, phenotypic background shapes responsiveness to OXT, underscoring the significance of strain-specific factors in interpreting neuromodulatory effects and motivating future investigations into the underlying receptor-level and circuit-based mechanisms.

**Institutional review board statement:** The animal study protocol was approved by the Ethics Committee of the Faculty of Biology, “Alexandru Ioan Cuza” University, Iasi, with registration no. 1349/20 March 2024.

## Supplemental data

Supplemental data are available at the following link: https://www.bjbms.org/ojs/index.php/bjbms/article/view/13819/4165.

## Data Availability

All data generated or analyzed during this study are included in this published article.
